# Biofluorescence imaging-guided implantoplasty for the management of peri-implantitis: a retrospective case series

**DOI:** 10.1186/s12903-025-07644-1

**Published:** 2026-01-13

**Authors:** Seo-Jun Lee, Jung-Hyun Kwon, Yong-Suk Choi, Pil-Young Yun, Jeong-Kui Ku

**Affiliations:** 1 Department of Oral and Maxillofacial Surgery, Section of Dentistry, Seoul National University Bundang Hospital, 300 Gumi-dong, Bundang-gu, Seongnam, Gyeonggi-do Republic of Korea; 2 Office of Human Resources, Development, Armed Forces Capital Hospital, Armed Forces Medical Command, Seongnam, Republic of Korea; 3 Department of Dentistry and Dental Research Institute, School of Dentistry, Seoul National University, Seoul, Republic of Korea

**Keywords:** Biofilm, Dental implants, Fluorescence imaging, Implantoplasty, Peri‑implantitis, Case series

## Abstract

**Background:**

Peri-implantitis is a biofilm-driven inflammatory condition that often requires surgical intervention. This case series aimed to evaluate the clinical outcomes of implantoplasty guided by a biofluorescence imaging system (BIS) in the surgical treatment of peri-implantitis.

**Methods:**

Seven patients (13 implants) with peri-implantitis underwent BIS-guided flap surgery and selective implantoplasty. Probing depth (PD) and bleeding on probing (BOP) were assessed at baseline, and at 1, 3, and 6 months postoperatively, as well as at the final follow-up. Nonparametric statistical analyses were used to evaluate longitudinal changes across time points.

**Results:**

A total of 7 patients (3 males and 4 females; mean age 71.9 ± 10.0 years) with 13 implants diagnosed with peri‑implantitis were included. The mean follow‑up period was 9.9 ± 2.7 months. The mean PD significantly decreased from 6.8 ± 1.5 mm at baseline to 3.0 ± 1.4 mm at the final follow‑up (*p* < 0.001), and BOP was completely resolved in all implants. All implants remained clinically stable, with no complications or recurrence.

**Conclusions:**

BIS enabled the accurate identification of mature biofilm and facilitated site‑specific, conservative decontamination. This selective approach, which targets infected surfaces while preserving implant structure, may be clinically advantageous for peri‑implantitis management. Further randomized controlled trials are warranted to validate these findings.

**Supplementary Information:**

The online version contains supplementary material available at 10.1186/s12903-025-07644-1.

## Background

Peri-implantitis is a pathological condition characterized by inflammation of the peri-implant mucosa, and its diagnosis is based on clinical signs such as bleeding on probing (BOP) and/or suppuration, increased probing depth (PD) compared to previous exams, and radiographic bone loss beyond normal remodeling after healing. When previous records are unavailable, PD ≥ 6 mm with bleeding/suppuration and bone loss ≥ 3 mm from the implant shoulder support the diagnosis. This framework helps distinguish peri-implantitis from peri-implant mucositis and guides treatment decisions [1]. 

With the increasing prevalence of dental implant therapy, the incidence of peri-implantitis has risen, posing a significant clinical challenge [2, 3]. Although a variety of therapeutic approaches—ranging from mechanical debridement to antimicrobial therapy and surgical intervention—have been proposed, no universally accepted protocol has been established, particularly for advanced cases with significant bone loss [4, 5]. 

Among the various surgical options, implantoplasty has emerged as a widely adopted resective technique for the Treatment of peri-implantitis [6, 7]. This procedure entails the mechanical decontamination and polishing of the exposed implant surface, thereby reducing surface roughness and limiting future microbial re-colonization [8, 9]. In fact, surface modification by implantoplasty or chemical decontamination has shown superior efficacy in reducing early biofilm formation compared to conventional debridement procedures [10]. However, aggressive implantoplasty may compromise the mechanical strength of the fixture by reducing its diameter, thereby increasing the risk of implant fracture [10]. It is therefore essential to selectively target contaminated implant surfaces while minimizing unnecessary structural alterations [11]. 

Recent advancements have enabled real-time intraoperative visualization of target lesions using BIS [12–15]. A modified BIS, Qray-Pen (AIOBIO, Seoul, Republic of Korea) emitting 405 nm visible blue light has been used to detect demineralization and necrotic tissues without requiring additional fluorophores [13]. Histologic validation of fluorescence patterns has been reported in various oral lesions, including necrotic bone following graft procedures and specimens from MRONJ surgeries [12–15]. This technology enhances the clinician’s ability to identify and remove infected tissues with greater precision [16]. In 2024, BIS-guided MRONJ surgeries demonstrated the successful preservation of adjacent teeth and implants by selectively removing only fluorescent, infected tissue—supporting a minimally invasive surgical paradigm [17, 18]. In these cases, red-fluorescent signals correlated with mature biofilm accumulation, and their targeted removal resulted in successful treatment outcomes [19]. Inspired by these findings, we hypothesized that BIS-guided implantoplasty may offer similar benefits for the surgical management of peri-implantitis. Accordingly, this study aims to report the clinical outcomes of BIS-guided implantoplasty in the treatment of peri-implantitis, focusing on its precision, safety, and potential for preserving implant integrity.

## Methods

This study received Institutional Review Board approval from Seoul National University Bundang Hospital (IRB No. B-2505-974-104). This retrospective, single-center, consecutive case series included 7 patients (13 implants) diagnosed with peri-implantitis who presented to the Department of Oral and Maxillofacial Surgery, Seoul National University Bundang Hospital, between April and July 2024. The inclusion criteria were as follows: (1) intraoperative use of Qray-Pen to guide implantoplasty; and (2) peri-implantitis defined by PD ≥ 6 mm with BOP and/or suppuration and radiographic bone loss consistent with the 2018 AAP/EFP framework; [1] (3) functional loading for ≥ 12 months; and (4) availability of postoperative clinical follow-up data beyond 3 months, including PD, BOP, suppuration, and periapical radiographs. Exclusion criteria were: uncontrolled systemic conditions, prior head and neck radiotherapy, other inflammatory bone diseases (e.g., osteomyelitis/osteonecrosis), implant fracture, and current smoking or a history of smoking within the past 6 months.

### Clinical measurements

PD and BOP were recorded at six sites per implant (mesiobuccal, mid‑buccal, distobuccal, mesiolingual/palatal, mid‑lingual/palatal, and distolingual/palatal) using a calibrated Marquis periodontal probe with gentle force (~ 0.25 N). For each implant and time points, the deepest PD among the six sites was analyzed. BOP was considered positive if bleeding occurred within 30 s after gentle probing, and suppuration was recorded as present or absent upon gentle probing or light pressure.

Standardized periapical radiographs were obtained at each follow‑up, and vertical bone levels were assessed relative to the implant shoulder using pixel‑to‑millimeter calibration. The prosthetic loading time point served as the radiographic baseline, thereby excluding physiologic crestal bone remodeling before prosthesis delivery or within the first year after placement. Any bone loss beyond this baseline was considered progressive; measurements were used to confirm its presence rather than for primary quantitative statistical analysis. Radiographic assessments were performed by multiple trained examiners following a standardized protocol. If a discrepancy of > 1 mm in PD was noted between examiners, the site was re-examined to establish a consensus value.

### Preoperative and maintenance care

Patients received plaque control instruction (PCI) at 6‑month intervals after implant placement; oral hygiene was re‑evaluated and instruction reinforced at each visit. Site‑specific scaling was performed when patients reported discomfort or when clinically indicated, and supportive implant therapy was scheduled at 3- to 6-month intervals according to individual risk. A preoperative scaling appointment was scheduled approximately 2 weeks prior to the implantoplasty procedure. Prostheses were not routinely removed during maintenance; occlusion and screw stability were checked when indicated.

### Biofluorescence imaging guided implantoplasty protocol

**Fig. 1:**
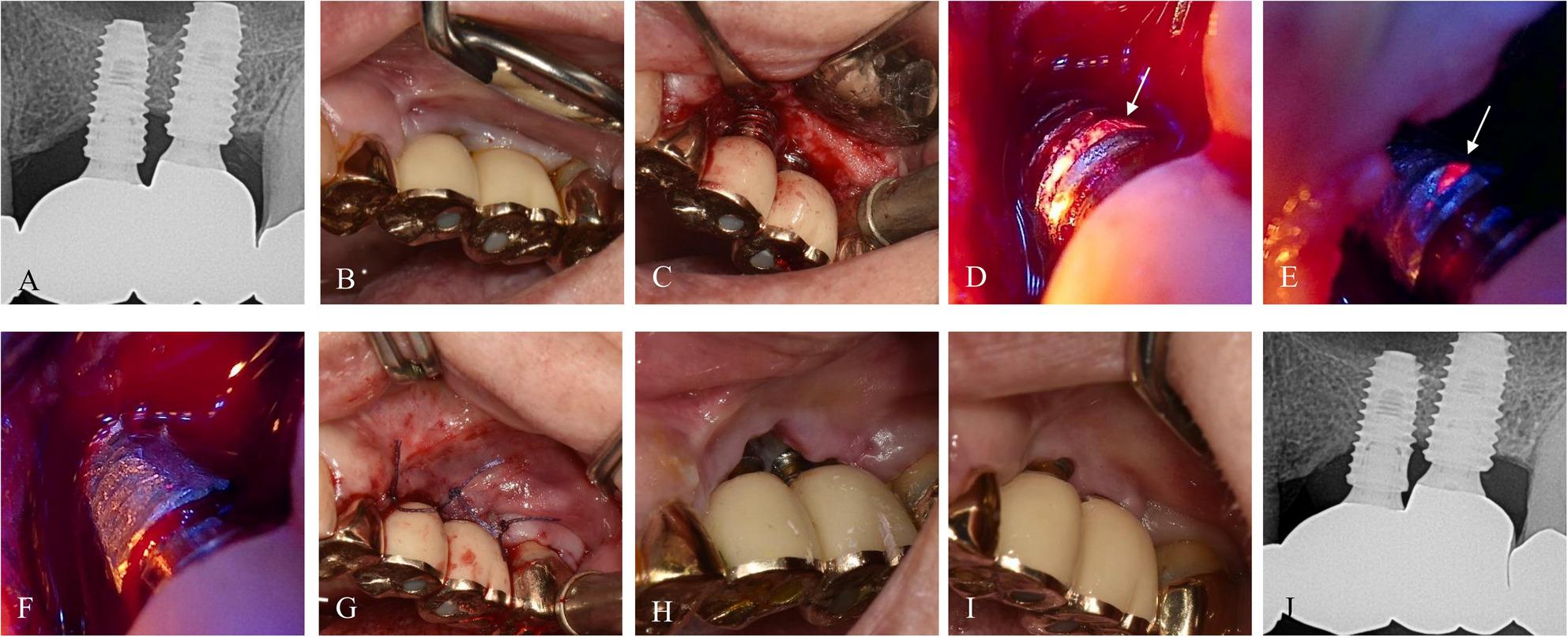
Case 1 (69-year-old female, #25i-26i) treated with BIS-guided implantoplasty. **A**, **B** Preoperative radiograph and intraoral image. **C** Modified Widman flap operation with curettage of peri-implant tissue. **D**-**G** Sequential BIS-guided fluorescence images during implantoplasty showing selective removal of the red-fluorescent area. **H**, **I** Postoperative intraoral images at 1 week and 13.4 months showing no recurrence of peri-implantitis or peri-gingivitis. **J** Radiograph at 13.4 months demonstrating stable bone levels

The surgical procedures were performed by an experienced oral and maxillofacial surgeon (J.K. Ku) according to minimally invasive principles, with biofluorescence guidance provided by the Qray‑Pen. All procedures were performed under local anesthesia. (Fig. 1) The surgical field was disinfected using 0.12% chlorhexidine gluconate. Prosthetic components were not removed during the procedure. A modified Widman flap was elevated, and granulation tissue was debrided using a Molt curette or Gracey curettes (Hu-Friedy, IL, USA). The field was irrigated with sterile saline, and hemostasis was achieved using pressure gauze.

A BIS, Qray-Pen emitting 405 nm blue light was applied in a darkened field to identify red fluorescence, indicative of mature bacterial biofilm. Implantoplasty was selectively performed only in fluorescent areas using a 2‑mm carbide round bur at 200,000 rpm under copious irrigation. Real-time BIS feedback guided the extent of surface modification, ensuring thorough removal of contaminated regions while minimizing unnecessary alteration of healthy implant threads. Titanium particles were carefully removed to prevent contamination of surrounding tissues.

Interrupted sutures were placed using 4 − 0 monofilament (BioTex; Purgo Biologics, Seongnam, Republic of Korea). Postoperatively, patients received amoxicillin (500 mg, bid for 5 days) and ibuprofen (600 mg, bid for 5 days). Chlorhexidine mouthwash (0.12%) was prescribed for twice-daily rinsing until postoperative day 10.

### Postoperative follow-up and evaluation

Sutures were removed on postoperative day 10. Clinical parameters, including PD, BOP, and suppuration, were assessed at 1, 3, and 6 months postoperatively, and PCI was performed at each visit [20]. Thereafter, follow-up and maintenance visits were scheduled at 3–6-month intervals, and standardized periapical radiographs were obtained to evaluate peri-implant bone levels. No participants were lost to follow‑up during the study period.

The primary outcomes were the reduction in PD and the resolution of BOP. Data normality was assessed using the Shapiro–Wilk test. As the PD data did not follow a normal distribution, comparisons between baseline and each postoperative time points were performed using the Wilcoxon signed-rank test (SPSS v25.0, IBM Corp., NY, USA). Statistical significance was set at *p* < 0.05.

This case series has been reported in line with the PROCESS 2025 guidelines; the completed checklist is provided as Supplementary File S1 (Additional file 1).

## Results

**Fig. 2:**
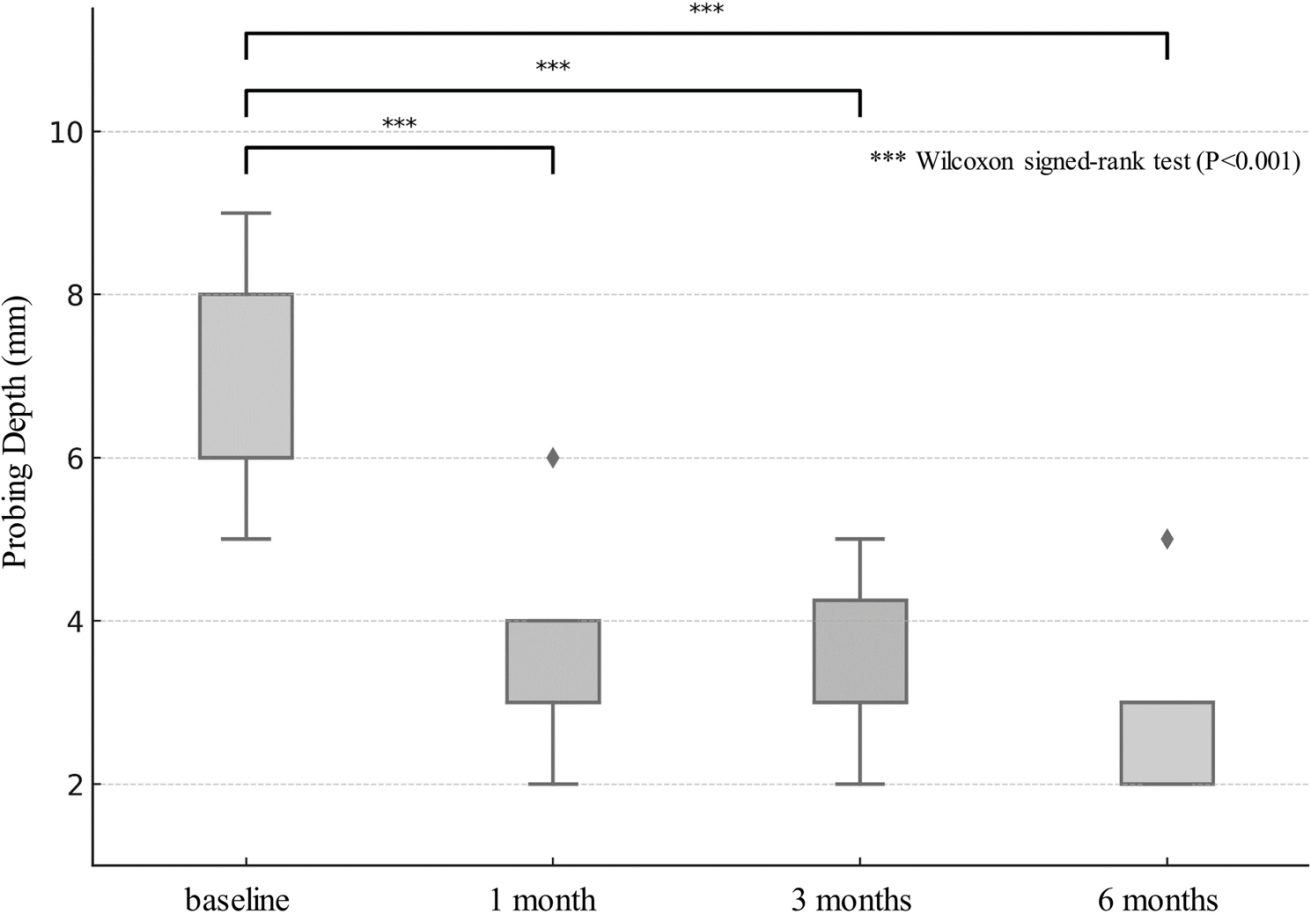
Probing depth at four time points: preoperative (baseline) and 1, 3, and 6 months postoperatively

A total of 7 patients (3 males and 4 females; mean age, 71.9 ± 10.0 years) with 13 implants diagnosed with peri-implantitis were included in this study. The mean functional duration of the implants prior to peri-implantitis surgery was 136.8 months (Table 1). At baseline, the mean PD was 6.8 ± 1.5 mm. Postoperative measurements demonstrated significant reductions; the mean PD was 3.36 ± 1.12 mm at 1 months, 3.64 ± 0.91 mm at 3 months, and 2.79 ± 0.43 mm at the final follow-up (Fig. 2). The differences between baseline and each postoperative time points were statistically significant (*p* < 0.001). At the final follow-up (mean:9.9 ± 2.7 months), all implants exhibited complete resolution of BOP. The final mean PD was 3.0 ± 1.4 mm.

**Table 1:** Table summarizing demographic information, medication history, implant site, surgical details, and clinical outcomes for the seven patients included in this study

Patient No.	1		2	3		4		5		6		7	
Age/Sex	69/F		76/F	59/M		61/M		87/M		71/F		75/F	
Systemic disease	Hypertension, Diabetes mellitus, hyperlipidemia, Depression		-	-		Snoring		-		Hypertension		Hyperlipidemia	
Location	#25i	#26i	#25i	#32i	#42i	#46i	47i	#16i	#17i	#12i	#14i	#12i	#13i
Prosthetic characteristics	25i-26i SB (PFG, SCRP)		Hyb-FA (SCRP)	32i-42i SB (MZr, SCRP)		SC (MZr, SCRP)	SC (PFG, CEM)	16i-17i SB (PFM, SCRP)		16i-26i FA (MZr, SCRP)		12i-22i SB (MZr, SCRP)	13i-17i SB (MZr, SCRP)
Functional period before implantoplasty (months)	119.4	119.4	96.3	53.9	53.9	160.6	218.6	89.8	89.8	173.3	173.3	133.2	133.2
Deepest probing depth at baseline (mm)	8	8	6	6	5	9	9	6	6	6	6	8	8
Bleeding on probing at baseline	+	+	+	+	+	+	+	+	+	+	+	+	+
Adjunctive agent for surgery	+	+	+	+	+	+	+	+	+	+	+	+	+
- Minocycline	+	+	-	-	-	-	-	-	-	+	+	+	+
- Collagen plug	-	-	-	+	+	-	-	+	+	-	-	-	-
- PDRN	-	-	+	+	+	+	+	+	+	-	-	-	-
Follow-up period (Months)	8.1	8.1	9.3	10.1	10.1	5.2	5.2	8.2	8.2	7.4	7.4	12.2	12.2
Deepest probing depth at final follow-up (mm)	3	3	3	3	3	7	7	4	4	3	3	3	3
Bleeding on Probing at final follow-up	-	-	-	-	-	-	-	-	-	-	-	-	-

*Abbreviations: SCRP* Screw‑retained, *CEM* Cement‑retained, *SC* Single crown, *SB* Splinted bridge, *FA* Full‑arch, *PFG* Porcelain‑fused‑to‑gold, *PFM* porcelain‑fused‑to‑metal, *MZr* Monolithic zirconia, *Hyb* Hybrid full‑arch. Sites use FDI numbering; “25i–26i bridge” denotes a splinted restoration across #25i–26i

Symbols: +, present; –, absent

### Case 1. A 69-year-old female with hypertension, diabetes mellitus, hyperlipidemia, and depression

The patient presented with peri‑implant discomfort and swelling around #25i–26i and reported food impaction. At baseline, the deepest PD was 8 mm with BOP (Fig. 1). BIS-guided flap surgery and implantoplasty were performed with local application of Minocure (minocycline hydrochloride, NIBEC, Jincheon, Republic of Korea). At the 7-month follow-up, supragingival scaling was performed. At 13.4-month follow-up, the deepest PD was < 4 mm with no BOP, and peri‑implant tissues appeared clinically healthy.

### Case 2. A 76-year-old female with no reported systemic diseases

**Fig. 3:**
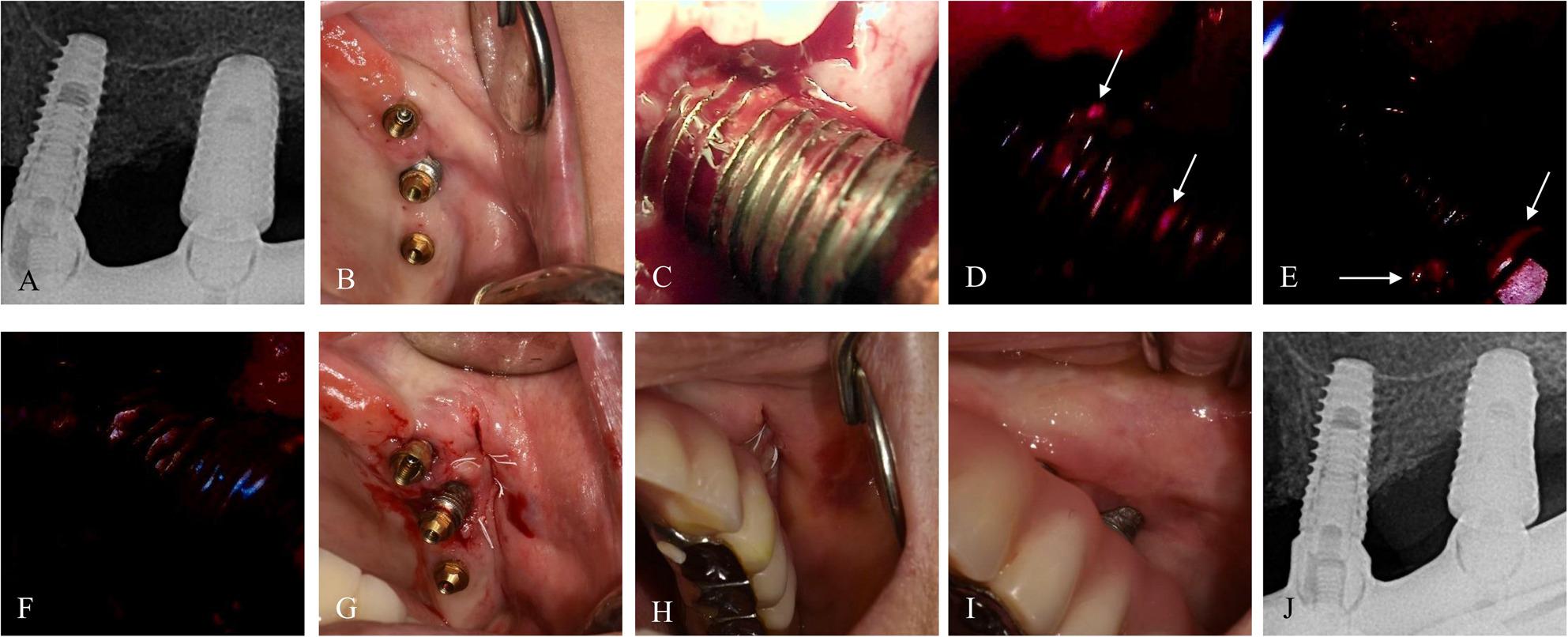
Case 2 (76-year-old female, #25i) treated with BIS-guided implantoplasty with adjunctive application of PDRN and vestibuloplasty. **A**, **B** Preoperative radiograph and intraoral image. **C**–**F** Sequential BIS-guided fluorescence images during implantoplasty. **G** Vestibuloplasty was performed after BIS-guided implantoplasty with primary closure. **H**, **I** Postoperative intraoral images at 1 week and 9.4 months. **J** Radiograph at 10.1 months demonstrating stable bone levels

The patient presented with peri‑implant discomfort near #25i and reported pain during tooth brushing. At baseline, the deepest PD was 6 mm with BOP (Fig. 3). BIS‑guided implantoplasty was performed with an adjunctive injection of Placentex (polydeoxyribonucleotide[PDRN]; Pharma Research Co., Seongnam, Republic of Korea) to promote soft tissue healing. Vestibuloplasty was then conducted using the modified Edlan-Mejchar technique. At the 3-month follow-up, symptoms had resolved. The deepest PD was 3 mm with no BOP, and the soft tissue was well‑regenerated. At the 9.3-month follow-up, mild gingival recession was noted, but the deepest PD remained < 3 mm with healthy gingiva.

### Case 3. A 59-year-old female with no reported systemic diseases

**Fig. 4:**
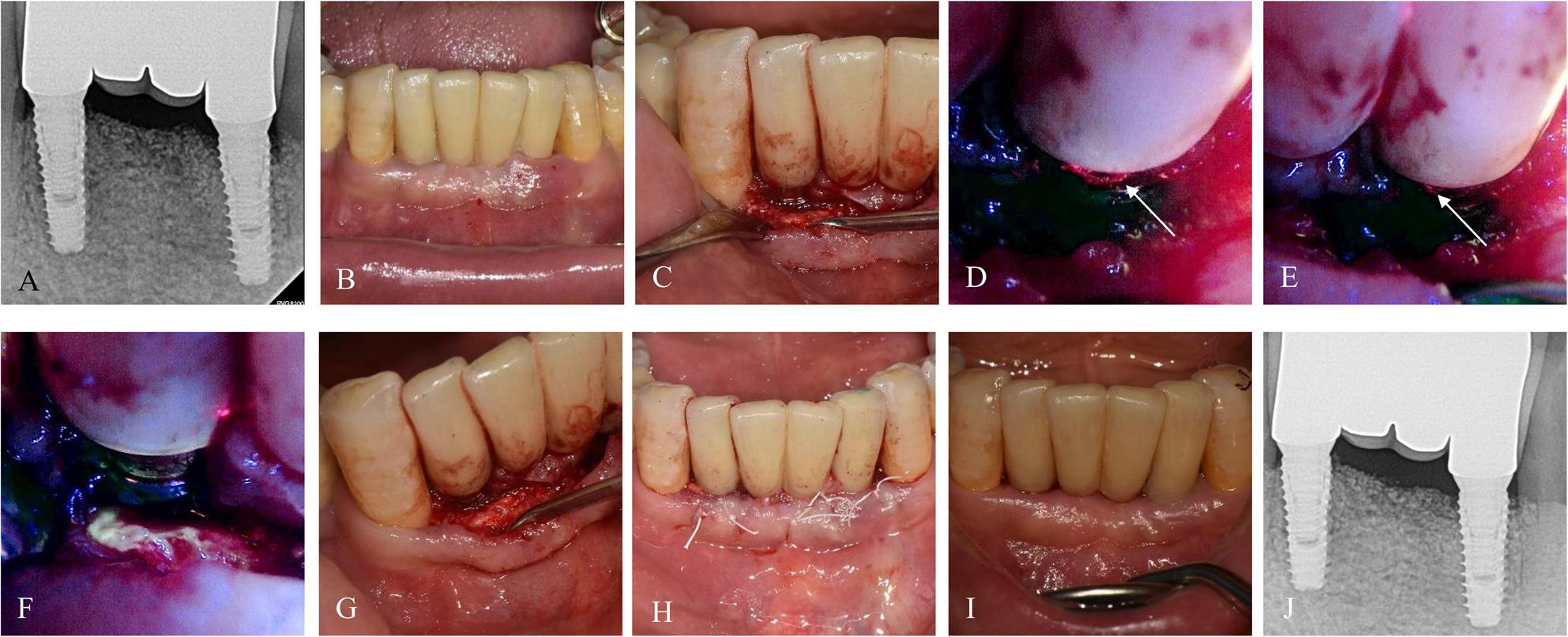
Case 3 (59-year-old female, #32i-42i) treated with BIS-guided implantoplasty on only #32i due to no red-fluorescence on #42i, with adjunctive application of PDRN and AteloPlug. **A**, **B** Preoperative radiograph and intraoral image. **C**–**H** BIS fluorescence-guided implantoplasty on #32i and modified flap surgery on #42i. **I** Postoperative intraoral images at 6.8 months. **J** Radiograph at 7.1 months demonstrating stable bone levels

The patient presented with peri‑implant discomfort and suppuration around #32i. At baseline, the deepest PD was 6 mm with BOP and pus discharge (Fig. 4). BIS-guided surgery revealed red fluorescence only on implant #32i, which underwent selective implantoplasty, while the adjacent, non-fluorescent implant #42i was preserved. After the procedure, adjunctive injection of PDRN and placement of AteloPlug (collagen plug, Bioland, Cheonan, Republic of Korea) were used for soft tissue healing. At the 3- and 6-month follow-ups, peri‑implant tissues were stable, with the deepest PD < 3 mm at #32i and < 2 mm at #42i. No recurrence of inflammation or infection was observed through 13.9 months.

### Case 4. A 61-year-old male with a history of snoring

**Fig. 5:**
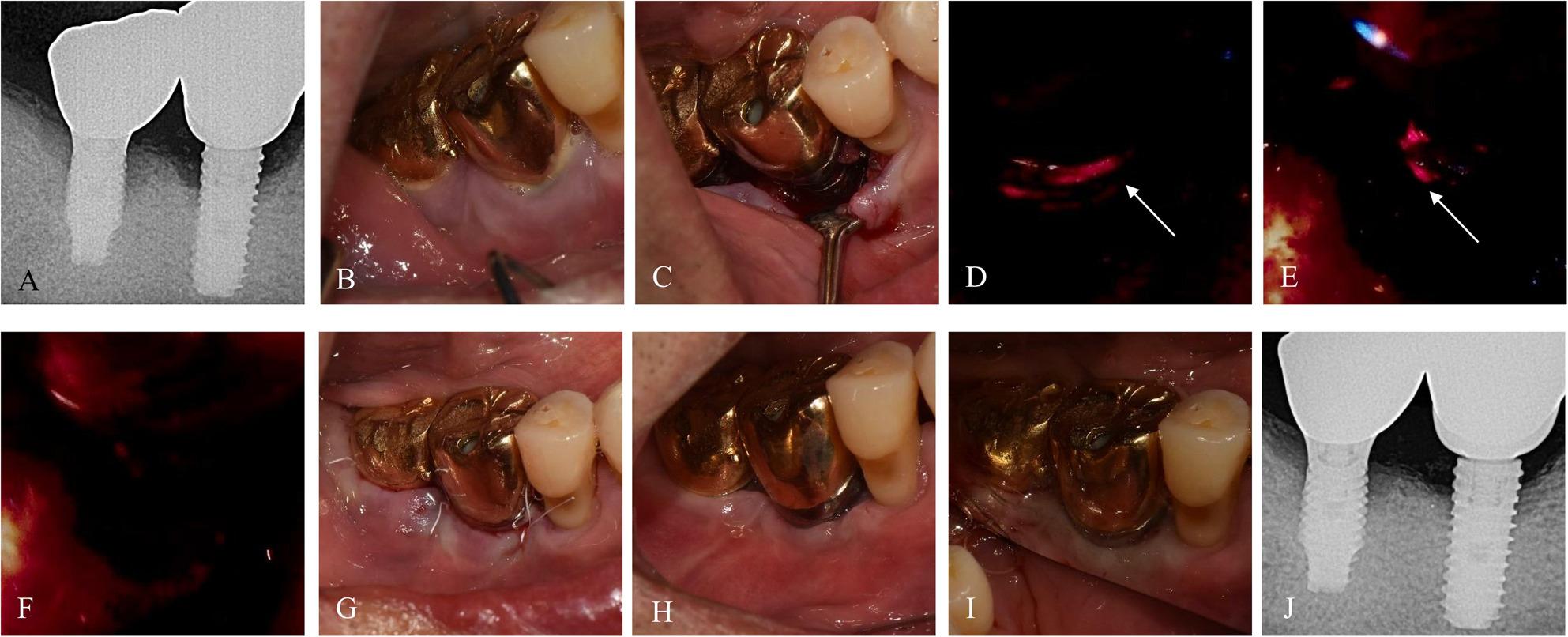
Case 4 (61-year-old male, #46i and #47i) treated with BIS-guided implantoplasty with adjunctive application of PDRN. **A**, **B** Preoperative radiograph and intraoral image. **C**–**G** Sequential BIS-guided fluorescence images during implantoplasty. **H**, **I** Postoperative intraoral images at 1.9 and 7.1 months. **J** Radiograph at 7.1 months demonstrating stable bone levels

The patient presented with peri‑implant discomfort and suppuration around #46i and #47i. At baseline, the deepest PD reached 9 mm with BOP (Fig. 5). BIS‑guided implantoplasty was performed with adjunctive PDRN injection around the gingiva. The deepest PD showed progressive improvement at each follow-up: it reduced to < 6 mm at 1 month, < 5 mm at 3 months, and < 3 mm at 7.1 months. No complications were observed throughout the follow-up period.

### Case 5. An 87-year-old male with no reported systemic diseases

**Fig. 6:**
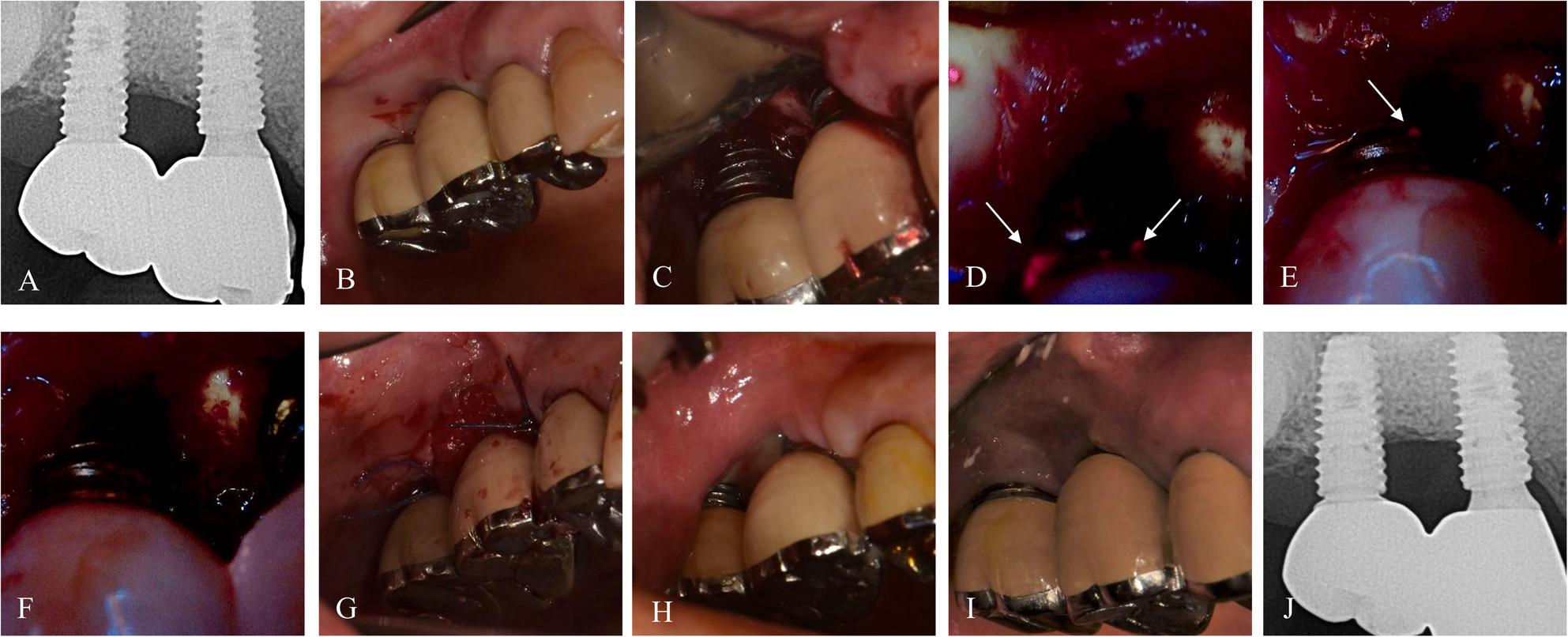
Case 5 (87-year-old male, #16i-17i) treated with BIS-guided implantoplasty with adjunctive application of PDRN and AteloPlug. **A**, **B** Preoperative radiograph and intraoral image. **C**–**G** Sequential BIS-guided fluorescence images during implantoplasty with AteloPlug. **H**, **I** Postoperative intraoral images at 1 week and 8.2 months. **J** Radiograph at 8.2 months postoperative demonstrating stable bone levels

The patient presented with recurrent peri‑implantitis involving #16i–17i. At baseline, the deepest PD was 6 mm with BOP (Fig. 6). BIS‑guided implantoplasty was performed with local injection of PDRN and placement of AteloPlug. At the 1-month follow-up, the deepest PD was < 3 mm bilaterally with no BOP. These clinical improvements were maintained at 3 and 6 months and persisted at 8.2 months, indicating long-term peri-implant health.

### Case 6. A 71-year-old female with hypertension

**Fig. 7:**
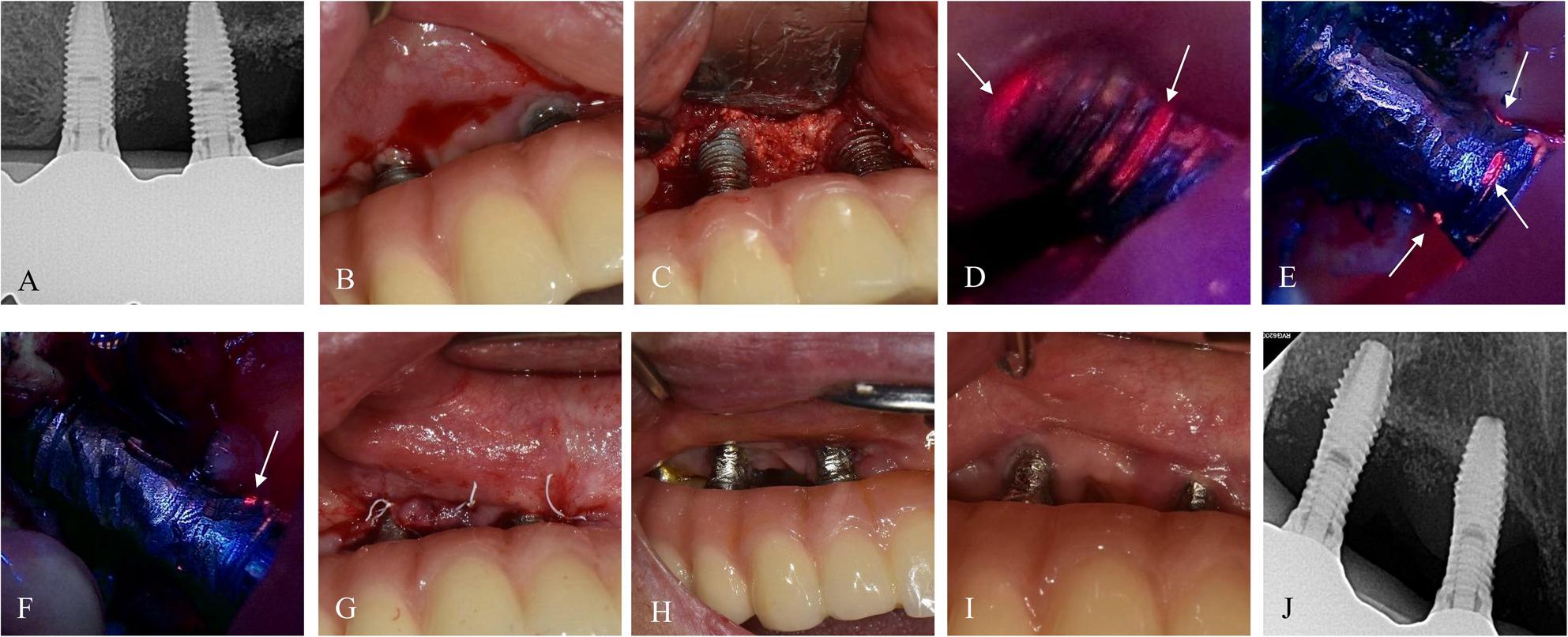
Case 6 (71-year-old female, #12i-14i) treated with BIS-guided implantoplasty with adjunctive application of Minocure. **A**, **B** Preoperative radiograph and intraoral image. **C**–**G** Sequential BIS-guided fluorescence images during implantoplasty (**H**, **I**) Postoperative intraoral images at 10 days and 11.8 months. **J** Radiograph at 11.8 months demonstrating stable bone levels

The patient presented with peri‑implant discomfort and suppuration around #12i-14i. At baseline, the deepest PD was < 6 mm with BOP and pus discharge (Fig. 7). BIS‑guided implantoplasty was performed with local application of Minocure. At 1-month follow-up, the deepest PD was < 3 mm with no BOP and remained stable throughout 11.8 months.

### Case 7. A 75-year-old female with hyperlipidemia

**Fig. 8:**
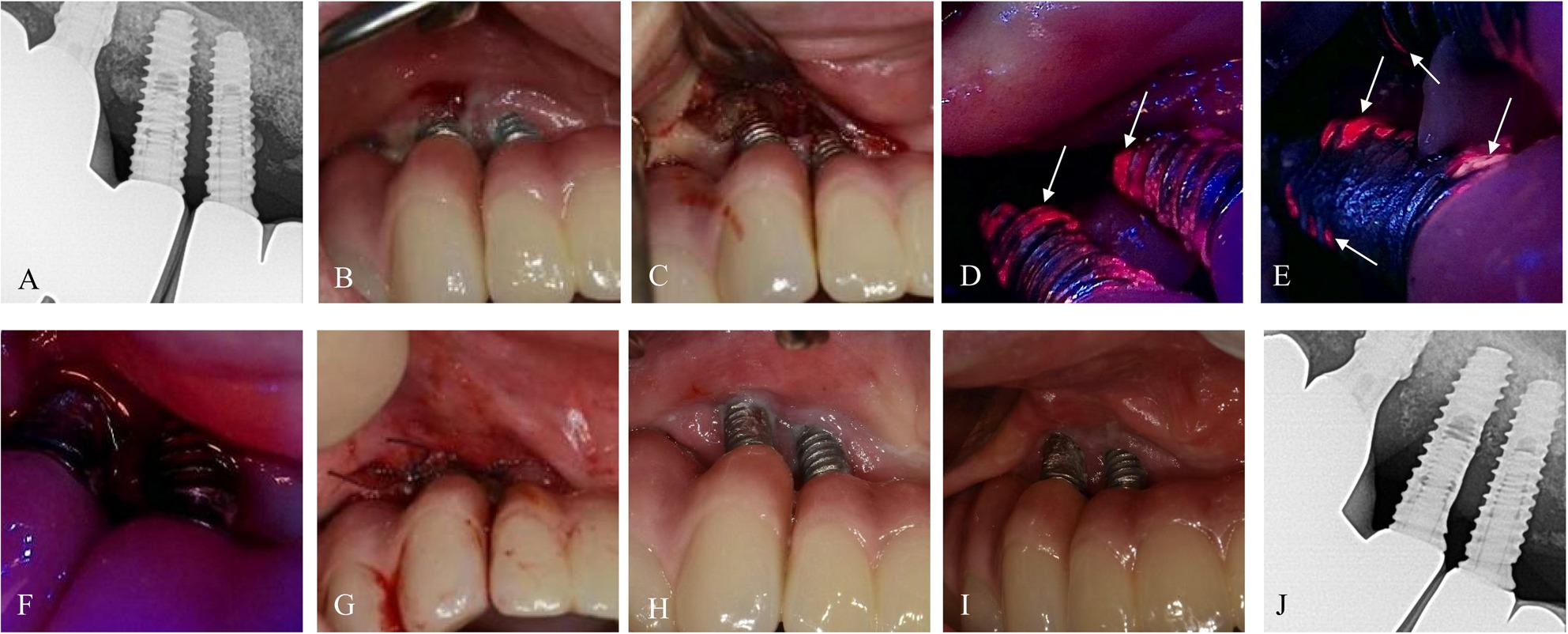
Case 7 (75-year-old female, #12i and #13i) was treated with BIS-guided implantoplasty with adjunctive application of Minocure, alveoloplasty, and vestibuloplasty. **A**, **B** Preoperative radiograph and intraoral image. **C**–**G** Sequential BIS-guided fluorescence images during implantoplasty and alveoloplasty. **H**, **I** Postoperative intraoral images at 2.4 and 4 months. **J** Radiograph at 13.4 months demonstrating maintained stable bone levels

The patient presented with right cheek tenderness and peri‑implant suppuration around #12i and #13i. At baseline, the deepest PD was 8 mm, with BOP and pus discharge (Fig. 8). BIS detected red fluorescence on the residual alveolus at #13i. BIS‑guided implantoplasty, alveoloplasty, and vestibuloplasty were performed with local application of Minocure. Postoperatively, symptoms resolved, and the deepest PD remained < 3 mm with no BOP throughout 13.4-month follow-up period.

## Discussions

This case series demonstrated the clinical outcomes of BIS-guided implantoplasty for the treatment of peri-implantitis. Among 7 patients (13 implants), a significant reduction in PD was observed, from 6.8 ± 1.5 mm preoperatively to 3.0 ± 1.4 mm at the mean 9.9 ± 2.7-month follow-up. BOP was entirely eliminated in all cases. These findings indicate that BIS-guided implantoplasty can be an effective surgical strategy for resolving peri-implantitis while maintaining implant function and stability.

While the ideal treatment strategy for peri-implantitis depends on the extent of inflammation and bone loss, no universally accepted protocol has been established to date, especially for advanced cases [6]. Implantoplasty has long been employed as an adjunctive resective technique for peri-implantitis, aiming to remove contaminated threads and smooth the implant surface to prevent bacterial recolonization [8, 9]. Mechanical decontamination of the implant surface has been shown to reduce biofilm formation and limit further bone loss, thereby improving implant survival [9]. Although the resulting smooth surface may promote bacterial colonization due to the loss of micro-retentive features [21], surface modification has also been reported to reduce early biofilm attachment compared with simple mechanical debridement [10]. 

Conventional implantoplasty typically achieves PD reductions of 2.5–3.1 mm and BOP improvement rates of 40–70% [22], whereas our series showed a greater mean PD reduction of 3.8 mm with complete elimination of BOP at a mean follow‑up of 9.9 months. According to Chan et al., regenerative approaches may result in 2.0–5.4 mm reductions in PD [23], and Tomasi et al. observed an average reduction of 2.8 mm at 12 months for both regenerative and resective therapies [24]. A 2019 RCT found that the use of Perio-Flow^®^ (EMS, Nyon, Switzerland) or Ti-brush^®^ (Straumann, Basel, Switzerland) led to greater PD reduction (2.2–2.4 mm) than plastic curettes (1.6 mm) [25]. Despite multiple etiological hypotheses, bacterial biofilm remains the most widely accepted cause of peri-implantitis [2, 4], and its elimination is the primary objective of treatment.

Traditional surgery often lacks the ability to precisely differentiate infected from healthy surfaces, leading to either insufficient decontamination or excessive surface removal, both of which can compromise outcomes. Implantoplasty also carries a mechanical risk: over-reduction of the implant diameter can significantly reduce fracture resistance, particularly in implants with external hex connections. Gehrke et al. reported a 32% reduction in implant resistance after implantoplasty [10]. Hence, limiting surface removal to infected areas while preserving structural integrity is critical. BIS-guided implantoplasty addresses these concerns by enabling real-time identification of mature biofilm via red fluorescence, allowing selective surface decontamination. This selective approach minimizes unnecessary surface modification and helps maintain the biomechanical stability of the implant. The technique aligns with the evolving paradigm of fluorescence-guided, minimally invasive surgery previously established in the treatment of medication-related osteonecrosis of the jaw (MRONJ) [14, 16, 18]. Andrade et al. first demonstrated that wide-field optical fluorescence can assist in visualizing peri-implant mucositis-associated biofilm [16], and recent studies further support the clinical relevance of BIS in guiding surgical debridement and improving outcomes.

The present findings can be interpreted in light of the underlying principles of BIS imaging. The reddish autofluorescence observed under 405‑nm illumination is generally attributed to porphyrin derivatives produced by mature anaerobic biofilms [26]. In vitro microcosm models have shown that fluorescence intensity increases with biofilm maturity, thickness, and bacterial load, supporting its clinical plausibility as a surrogate marker for surface contamination [27]. However, signal characteristics may vary depending on microbial composition, substrate properties, and imaging conditions, which emphasizes the necessity of integrating fluorescence findings with established clinical indicators such as PD, BOP, and radiographic assessment [27]. Previous translational studies have documented that peri‑implantitis lesions often exhibit fluorescence patterns correlated with contaminated implant threads, and that fluorescence‑guided debridement can improve treatment outcomes compared with white‑light inspection alone [28]. Moreover, the concept parallels fluorescence‑guided surgery in MRONJ, where auto-fluorescence enables selective removal of diseased tissue while preserving unaffected structures [18]. In support of pathologic specificity, histologic validation studies in MRONJ have indicated that hyper‑red fluorescent bone corresponds to inflamed tissue with bacterial invasion and osteolysis, whereas non‑red fluorescence corresponds to sclerotic, lamellar vital bone, suggesting that fluorescence patterns align with underlying histopathology and can aid intraoperative margin determination [13]. Nevertheless, BIS does not enable species‑level microbial identification, and its diagnostic reliability can be influenced by calibrated and consistent acquisition parameters for fluorescence detection, highlighting the importance of further methodological refinement [29, 30]. 

Taken together, our results suggest that BIS‑guided implantoplasty, enabled by real‑time visualization of mature biofilm, may balance effective surface cleaning with biomechanical preservation. Adequate decontamination and defect management were achieved via flap elevation without prosthesis removal, consistent with prior evidence showing no significant difference in radiographic bone fill between prosthesis‑removal and prosthesis‑maintenance protocols after regenerative surgery [31]. From a resource‑use perspective, although this workflow entails upfront device acquisition costs, it may shorten chair time during implantoplasty by enabling site‑specific decontamination and reducing the likelihood of repeat procedures. No formal cost‑effectiveness analysis was undertaken in this series, so these economic implications should be interpreted cautiously and confirmed prospectively.

In addition to BIS-guided implantoplasty, several adjunctive measures (e.g., locally delivered minocycline, PDRN, and collagen plugs) were applied at the clinician’s discretion Although these variables were not controlled, it is important to note that rigorous plaque control remains the cornerstone of peri-implantitis management. Within this framework, BIS-based biofluorescence imaging, compared with white-light inspection, offers more objective identification of mature biofilm and thereby enables site-specific, conservative surface decontamination—an element likely underpinning the observed clinical improvements. Nonetheless, given the retrospective design and the concomitant use of adjunctive agents, the independent effect attributable to BIS cannot be fully isolated. Building on these findings, we plan a prospective study that will standardize BIS-guided implantoplasty as the index intervention and establish a protocol for regenerative therapies (e.g., minocycline, PDRN, collagen plugs, bone grafts, connective tissue grafts, and free gingival grafts) to minimize confounding and permit stratified or adjusted analyses.

In this context, despite these promising results, the present study has several limitations. Foremost among these is the heterogeneity of the treatment protocol, including variations in adjunctive agents. Because these adjunctive measures were not standardized, the potential influence of these therapies on the observed effects cannot be ruled out, making it difficult to attribute the outcomes solely to BIS guidance. In addition, this study was conducted as a retrospective case series at a single institution with a small sample size; therefore, the findings should be regarded as exploratory and hypothesis‑generating, providing preliminary evidence of clinical feasibility rather than definitive proof of effectiveness. Although cases were collected consecutively to minimize selection bias, the observational design still yields a lower level of evidence compared with randomized controlled trials. Moreover, the short follow‑up period, absence of a control group, and further potential confounding from uncontrolled adjunctive therapies limit the strength of the conclusions. To better define the independent contribution of BIS, future research should be conducted as multicenter randomized controlled trials with standardized adjunctive protocols and include three‑dimensional bone analysis.

Another limitation is the lack of quantitative radiographic assessment of peri‑implant bone‑level changes. Although standardized periapical radiographs were obtained, this retrospective series did not include volumetric analysis or calibrated linear measurements. As a result, objective evaluation of bone fill or radiographic stability was not possible. In addition, the width of keratinized mucosa (KM) was not measured, which may have influenced the assessment of peri‑implant tissue health. Future studies should incorporate standardized, quantitative radiographic protocols—including CBCT‑based volumetric analysis—together with comprehensive soft‑tissue evaluation.

Notwithstanding these limitations, this study has notable strengths. First, consecutive recruitment of all eligible patients during the study period minimized the risk of selection bias. Second, all surgeries were performed by a single highly experienced operator, reducing inter‑operator variability. Third, real‑time BIS guidance was applied consistently for site‑specific decontamination across all cases, ensuring procedural uniformity. These factors may enhance the internal validity of the findings; however, the limitations noted above should be considered when interpreting the results.

## Conclusions

This case series demonstrated that BIS‑guided implantoplasty enables effective and selective decontamination of peri‑implantitis lesions by accurately identifying sites of mature biofilm. Significant clinical improvements, such as a reduction in PD and the resolution of BOP, were achieved in all cases without compromising implant integrity. Within the limitations of this small, single‑center case series, these findings suggest that BIS guidance may be a valuable adjunct in the surgical management of peri‑implantitis, and they warrant confirmation through larger-scale, controlled trials.

## Supplementary Information

Supplementary Material 1

Supplementary Material 2

## Data Availability

The datasets used and/or analyzed during the current study available from the corresponding author on reasonable request.

## Abbreviations

BIS: Biofluorescence imaging system
PDRN: Polydeoxyribonucleotide
