# Supplementary material for: Biofluorescence imaging-guided implantoplasty for the management of peri-implantitis: a retrospective case series
Source: BMC Oral Health. 2026 Jan 13;26:145. doi: 10.1186/s12903-025-07644-1 (PMC12829038; doi:10.1186/s12903-025-07644-1)
Supplement: Supplementary file 2 — Supplementary Material 2 [file 12903_2025_7644_MOESM2_ESM.docx]

Supplementary table 1. Table summarizing the probing depths at baseline and final follow-up for each implant site and surface in the seven patients included in this study.

| Patient No. | 1 | | 2 | 3 | | 4 | | 5 | | 6 | | 7 | |
| --- | --- | --- | --- | --- | --- | --- | --- | --- | --- | --- | --- | --- | --- |
| Location | #25i | #26i | #25i | #32i | #42i | #46i | 47i | #16i | #17i | #12i | #14i | #12i | #13i |
| Deepest probing depth at baseline (mm) | 8 | 8 | 6 | 6 | 5 | 9 | 9 | 6 | 6 | 5 | 5 | 8 | 8 |
| Mesiobuccal | 4 | 8 | 4 | 6 | 5 | 6 | 9 | 3 | 6 | 4 | 5 | 7 | 8 |
| Mid-buccal | 5 | 6 | 5 | 4 | 4 | 5 | 5 | 4 | 4 | 4 | 5 | 6 | 6 |
| Distobuccal | 8 | 5 | 6 | 4 | 3 | 9 | 6 | 6 | 6 | 5 | 5 | 8 | 6 |
| Mesiolingual/palatal | 4 | 6 | 4 | 4 | 4 | 5 | 6 | 4 | 5 | 3 | 5 | 6 | 7 |
| Mid-lingual/palatal | 5 | 4 | 5 | 3 | 3 | 6 | 4 | 3 | 4 | 4 | 4 | 5 | 5 |
| Distolingual/palatal | 6 | 4 | 5 | 4 | 3 | 8 | 4 | 5 | 5 | 4 | 5 | 6 | 6 |
| Deepest probing depth at final follow-up (mm) | 4 | 4 | 3 | 3 | 3 | 3 | 3 | 3 | 4 | 3 | 3 | 3 | 3 |
| Mesiobuccal | 3 | 4 | 3 | 3 | 3 | 3 | 3 | 3 | 4 | 3 | 3 | 3 | 3 |
| Mid-buccal | 3 | 3 | 3 | 2 | 2 | 3 | 2 | 3 | 3 | 2 | 3 | 3 | 2 |
| Distobuccal | 4 | 3 | 3 | 2 | 3 | 3 | 3 | 3 | 4 | 3 | 3 | 3 | 3 |
| Mesiolingual/palatal | 3 | 4 | 3 | 3 | 3 | 3 | 3 | 3 | 4 | 3 | 3 | 3 | 3 |
| Mid-lingual/palatal | 3 | 2 | 2 | 2 | 2 | 3 | 3 | 2 | 3 | 3 | 3 | 2 | 2 |
| Distolingual/palatal | 4 | 3 | 3 | 3 | 3 | 2 | 3 | 3 | 3 | 3 | 3 | 3 | 3 |
